# MiR-128-2 inhibits common lymphoid progenitors from developing into progenitor B cells

**DOI:** 10.18632/oncotarget.8161

**Published:** 2016-03-17

**Authors:** Yi Yang, Jie Xu, Huo Chen, Xia Fei, YuXu Tang, Yunqiu Yan, Huimin Zhang, Jinping Zhang

**Affiliations:** ^1^ Institutes of Biology and Medical Sciences, Soochow University, Suzhou, Jiangsu Province, People's Republic of China

**Keywords:** miR-128-2, CLP, B cell development, apoptosis, Immunology and Microbiology Section, Immune response, Immunity

## Abstract

A considerable number of studies revealed that B cell development is finely regulated by transcription factors (TFs). Recent studies suggested that TFs are coordinated with microRNAs to control the development of B cells in numerous checkpoints. In the present study, we first found that miR-128-2 was differentially expressed in various immune organs and immunocytes. B cell development was inhibited in miR-128-2-overexpressed chimera and transgenic (TG) mice in bone marrow with decreased preproB, preB, proB, immature B, and recirculating B cells, as well as increased common lymphoid progenitors (CLPs). Further experiments showed that the apoptosis of CLP decreased, but proliferation was not altered in miR-128-2-overexpressed mice. Extensive studies suggested that the inhibition of apoptosis of CLP may be caused by miR-128-2 targeting A2B and MALT1, thereby increasing the phosphorylation of ERK and P38 MAPK. Such findings have prompted future investigations on the function of miR-128-2 in lymph genesis.

## INTRODUCTION

B cells are derived from pluripotent hematopoietic stem cells (HSCs) in fetal liver and bone marrow (BM) after birth. The BM contains all stages of B cells from early stage common lymphoid progenitor (CLP) cells to mature B cells. B cell development is a highly regulated process that has been thoroughly investigated and characterized over the past decades because of well-characterized cell surface markers that can possibly define and purify distinct intermediates [1-4]. Previous studies have revealed the important transcription factors (TFs) in this development pathway, including early B cell factor-1 (EBF1), paired box protein 5 (PAX5), Ikaros, E box binding protein 2A (E2A), Notch1, FOXO1, and PU.1 [5]. With the progress of our understanding in gene regulation by TFs in B cell development, the regulatory networks between TFs and microRNAs (miRNAs) have attracted the interest of biologists [6].

MiRNAs are a class of small, noncoding RNAs with 18–24nt, which downregulate target genes at the posttranscriptional level. Majority of miRNA genes are transcribed by RNA polymerase II into long primary (pri) miRNA transcripts, which are then processed by the nuclear nuclease, Drosha, into ~60bp hairpins. Precursor (pre) miRNAs or pre-miRNAs are further cleaved in the cytosol by the Dicer nuclease into mature miRNAs. Mature miRNAs are then incorporated into the RNA-induced silencing complex in which they exert posttranscriptional repression of target mRNAs, either by inducing mRNA cleavage for degradation or by blocking mRNA translation [7-10].

The roles of miRNAs in B cell development and differentiation have been extensively explored for the past two decades. Bartel's group first demonstrated that miR-181a is differentially expressed in T and B cells. Ectopic expression of miR-181a in HSCs results in an increase in B cells both *in vivo* and *in vitro* [11]. In 2007, Rajewsky and Lodish found that miR-150 plays a pivotal role in B cell maturation. Deficiency of miR-150 leads to B1 cell expansion and enhances the humoral immune response. By contrast, the overexpression of miR-150 inhibits the transition of proB to preB by targeting c-myb translation [12, 13]. In the same year, several groups found that the depletion of miR-155 leads to impaired humoral response, resulting in reduced numbers of germinal center (GC) B cells and reduced amounts of secreted switched antigen-specific antibodies [14-16]. MiR-125b was also shown to inhibit plasma B cell differentiation and Ig secretion [17]. In 2010, Baltimore and his colleagues found that the overexpression of miR-34a in BM cells promotes the increase in the proportion of pro-B cells and decreases the number of pre-B cells by targeting the TF Foxp1, which is critical in the development of B cells [18]. Recently, Ramiro et al. found that overexpression of miR-217 in B cells enhances T cell-dependent immunization responses by improving the efficiency of GC formation, CSR, and SHM, as well as the generation of plasma and terminally differentiated memory B cells [6]. Hardy and colleagues identified the TF Arid3a as a key target of let-7; its ectopic expression is sufficient to induce B1 cell development in pro-B cells and silencing by knockdown blocks B1 development in fetal pro-B cells [19]. Broad depletion of total miRNA in the earliest stage or later stage of B cells by specific knockout of Dicer, which is essential for miRNA production, shows that miRNAs are key regulators for B cell development and activation. MiRNAs are involved in almost all checkpoints of B cell development and activation [20-22]. However, whether miRNAs are also involved in the transformation of CLPs to B cells remains unclear.

In this study, we first found that miR-128-2 was differentially expressed in B cells at different stages of development from CLP to mature B cells. By establishing the miR-128-2-overexpressed chimera and TG mice models, we found that miR-128-2-overexpressed mice showed a reduction in preproB, proB, preB, and immature B cells in the BM. Further studies suggested that miR-128-2 overexpression did not alter the proliferation or apoptosis of preproB, proB, and preB, but inhibited CLP to develop into preproB cells, partially caused by blocking the apoptosis of CLP. Further experiments demonstrated that miR-128-2 might exert this function by targeting A2B and MALT1, thereby affecting the phosphorylation of ERK and p38 MAPK.

## RESULTS

### MiR-128-2 was differentially expressed in various immune organs and immunocytes

**Figure 1:**
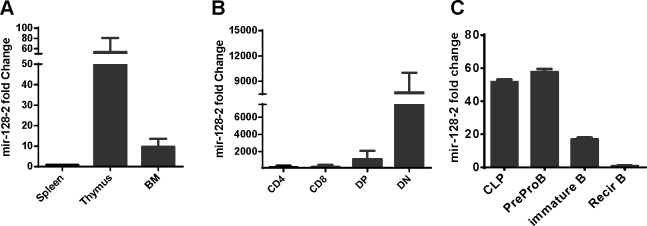
Expression of miR-128-2 in different immune organs **A.** and immunocytes **B.**, **C.** detected by real-time PCR. CD4 and CD8 single positive T cells were purified from spleen by using microbeads (Miltenyi Biotec Technology & Trading (Shanghai) Co., Ltd. Shanghai, China). DP and DN thymocytes were sorted from thymus by FACS Sorting. CLP, preproB, immature B and recirculating B (recirB) were sorted from BM by FACS sorting. The data represent three repeats.

To explore the function of miRNAs in the development of immunocytes, we first detected the expression profiles of miRNAs in some purified immunocytes (including BM monocytes, preproB cells, DN and DP thymocytes, CD4 and CD8 single-positive cells, and CD4^+^CD25^+^ regulatory T cells) by microarray. The heat map in Supplementary Figure 1 shows that miR-128 was highly expressed in DP thymocytes relative to other detected cells, which aroused our curiosity in the function of miR-128-2 in the development of immunocytes. To further verify the microarray data, we prepared total RNA from organs (including BM, thymocytes, and spleen) and purified lymphocytes (including DP and DN thymocytes from thymus, CD4^+^ and CD8^+^ single-positive T cells from spleen, CLP, preproB, immature B cell, and recirculating B cells from BM) to measure miR-128-2 expression by real-time PCR. As shown in Figure 1, miR-128-2 expression was higher in central immune organs (BM and thymus) compared with that in the spleen (Figure 1A) and then decreased progressively as T or B cells developed (Figure 1B and 1C). These data suggested that miR-128-2 may be involved in lymphocyte development.

### MiR-128-2 overexpression leads to inhibition of B cell development

**Figure 2:**
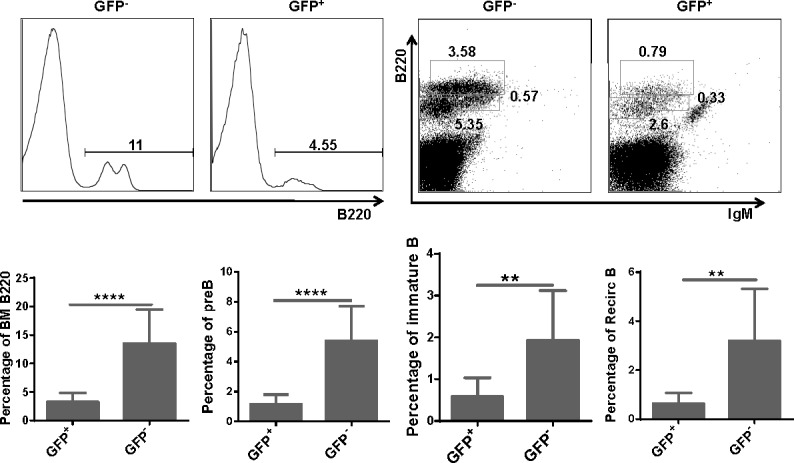
Total B cells and B cell subsets were reduced in BM of miR-128-2 overexpressed chimera mice B220^low^IgM^−^ cells represents preB cells, B220^low^IgM^+^ cells represents immature B cells, B220^high^IgM^+^ cells represents recirculating B cells (RecirB). The data represent five repeats. **P < 0.01, ****P < 0.0001.

To investigate whether upregulated expression of miR-128-2 can alter the development of lymphocytes, we adopted the miR-128-2-overexpressed chimera and TG mice models. After confirming the successful overexpression of miR-128-2 in 293T cells and chimera mice by real-time PCR or Northern blot (Supplementary Figure 2A, 2B), we prepared single-cell suspensions from BM, spleen, and thymus of two- to three-month-old chimera mice for flow cytometric analysis. We found that the percentages of total T cells and all T cell subsets, including DN, DP, CD4, or CD8 SP, were similar between WT and miR-128-2-overexpressed chimera mice (Supplementary Figure 3). However, the percentages of total B220^+^ cells and B cell subsets, including preproB (B220^+^IgM^−^), immature B, and recirculating B cells, were significantly reduced in miR-128-2-overexpressed chimera mice compared with those in WT mice (Figure 2).

**Figure 3:**
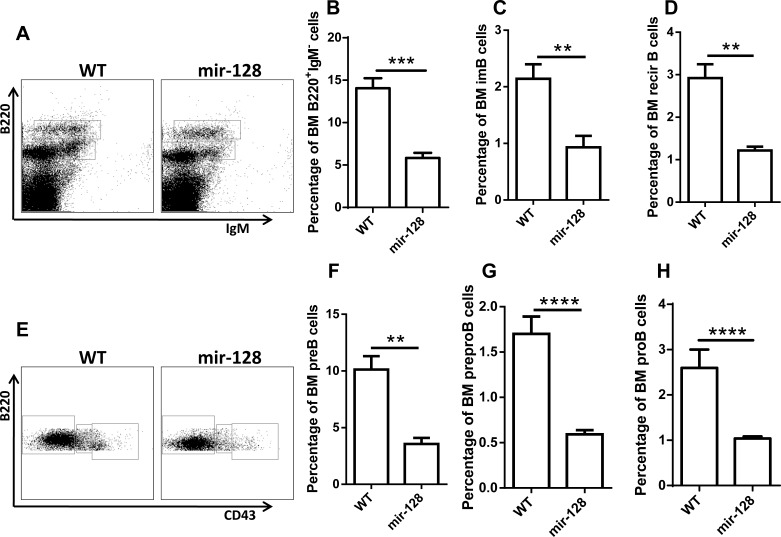
MiR-128-2-overexpressed TG mice have reduced total B cells and B cell subsets, including preproB, proB, preB, immature B, and recirculating B cells BM from 6-8 week-old WT or miR-128-2 TG mice were prepared and stained with relative antibodies followed by FACS analysis. **A.**- **D.** B220^low^IgM^+^, B220^high^IgM^+^ cells represent preB, immature B and recirculating B cells (RecirB) respectively, B220^low^IgM^−^ **A.** cells were gated and further analyzed with B220 and CD43 **E**.-**H**., B220^+^IgM^−^CD43^−^, B220^+^IgM^−^CD43^int^, B220^+^IgM^−^CD43^high^ cells represent preB, proB and preproB cells respectively. **P < 0.01, ***P < 0.001, ****P < 0.0001. The data represent five repeats.

To further confirm the phenotypes in chimera mice, we generated the miR-128-2 TG mice as described in the Materials and Methods. Supplementary Figure 2C indicated that miR-128-2 was successfully overexpressed in miR-128-2 TG mice. FACS analysis revealed that miR-128-2 TG mice displayed similar phenotypes to those in miR-128-2 chimera mice, that is, reduction in B220^+^IgM^−^ B cells (including preproB, preB and proB cells), immature B cells, and recirculating B cells in BM, without changes in T cells, cDCs, and MDSCs (Figure 3 and Supplementary Figure 4). Meanwhile, the periphery B cells including total B220^+^ B cells, marginal zone B cell (MZ) and follicular B cells (FO) were also reduced (Supplementary Figure 5A-5C), but the ratio of FO or MZ in B220^+^ cells was not changed (Supplementary Figure 5D, 5E). These results strongly suggested that miR-128-2 inhibited the development of B cells.

### MiR-128-2 prevents CLPs from developing into preproB cells

**Figure 4:**
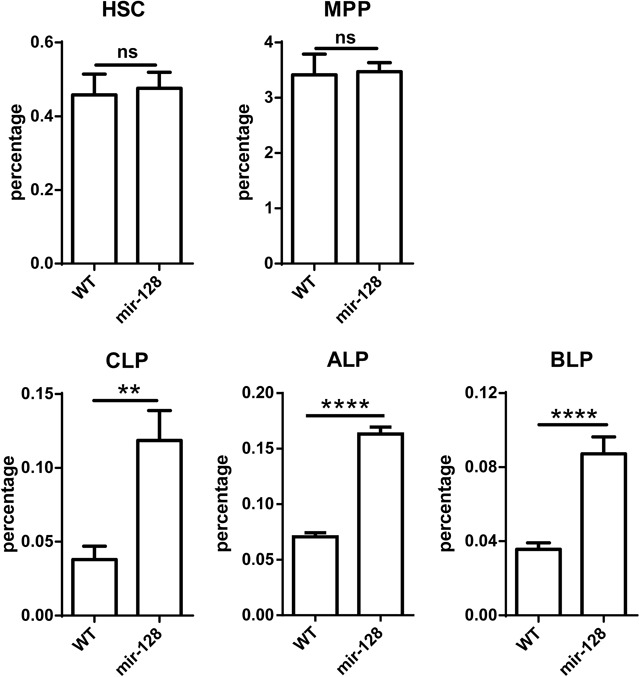
MiR-128-2 did not alter the percentages of HSC and MPP, but increased the percentages of CLP, ALP, and BLP compared with those in WT mice BM from 6-8 week-old WT or miR-128-2 TG mice were prepared and stained with relative antibodies (as described in M&M) followed by FACS analysis. The data represent five repeats. ns: no significant difference, **P < 0.01, ****P < 0.0001.

**Figure 5:**
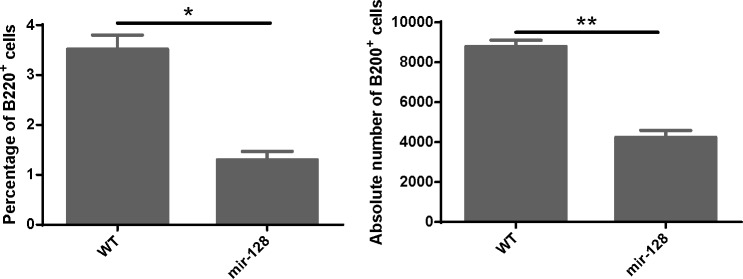
MiR-128-2-overexpressed CLP developed less B cells in the *in vitro* B cell culture system CLP cells were sorted from WT or miR-128-2 TG mice and cultured as described in M&M. The cultured cells were harvested and stained with B220 fluorescence antibody and analyzed with FACS. The absolute number of B220^+^ cells were calculated (total harvested cells×percentage of B220^+^ cells). The data represent three repeats. *P < 0.05, **P < 0.01.

B lymphocytes differentiate from HSCs through a series of well-characterized stages before peripheral migration. To determine which checkpoint miR-128-2 affects during B cell development, we compared HSC, MPP, and CLP levels between WT and miR-128-2-overexpressed TG mice. The results showed that the percentage of CLP was higher in miR-128-2 TG mice than that in WT mice without significant changes in earlier cells, such as HSCs and MPPs (Figure 4). CLP is composed of ALP and its progeny BLP [23]. Further analysis showed that both ALP and BLP increased in miR-128-2-overexpressed TG mice without alteration in their ratio in CLP (Figure 4 and Supplementary Figure 6). To verify whether the reduction of preproB, proB and preB cells is possible due to miR-128-2 affecting these cells themselves, we further analyzed the ratio of preproB, proB and preB cells in B220^+^IgM^−^ B cells in BM, the results showed that the ratio of preproB, proB and preB cells in B220^+^IgM^−^ B cells between WT and miR-128-2 TG mice was not significantly changed (Supplementary Figure 7). Given that miR-128-2 was overexpressed in all the body cells of TG mice, the possibility that the effect of miR-128-2 on B cell development might be indirectly through other cells must be considered. To test this hypothesis, we sorted CLPs from BM of miR-128-2 TG mice or WT mice by FACS and observed cell differentiation in an *in vitro* culture system as described in the literature [24, 25]. FACS analysis suggested that CLPs from miR-128-2 mice developed less B cells compared with those from WT mice (Figure 5). These data strongly suggested that overexpressed miR-128-2 directly blocked CLP from developing into preproB cells.

### MiR-128-2 inhibits the apoptosis of CLP

**Figure 6:**
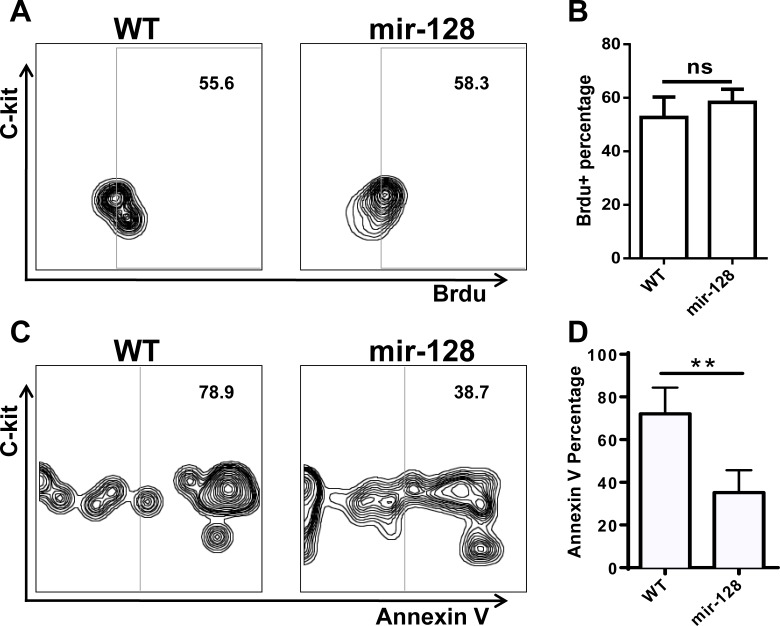
Annexin V staining and BrdU incorporation assay revealed that overexpressing miR-128-2 inhibited the apoptosis of CLP, but did not affect the proliferation of CLP BM cells were stained with relative antibodies for CLP as described in M&M, and gated Lin^−^c-kit^int^Sca1^+^ cells as CLP for Brdu **A.**, **B.** or Annexin V **C.**, **D.** analysis. The data represent three repeats. ns: no significant difference, **P < 0.01.

To explain the increase in CLP in miR-128-2-overexpressed mice, we measured the proliferation or self-renewal and apoptosis of CLPs by BrdU incorporation and annexin V staining, respectively. No difference in BrdU incorporation between WT and miR-128-2-overexpressed CLP (Figure 6A, 6B) was found. However, the frequency of apoptotic cells in miR-128-2-overexpressed CLP was lower than that in WT CLP (Figure 6C, 6D). To eliminate the possibility that the decrease in preproB cells was due to miR-128-2 affecting the preproB cells themselves, we detected the proliferation and apoptosis of preproB cells via BrdU and annexin V staining. Supplementary Figure 8 and 9 shows that miR-128-2 did not change the proliferation and apoptosis of preproB cells, thereby indicating that decreased apoptosis was responsible for the increase in CLP in miR-128-2-overexpressed mice.

### Assessing the target genes of miR-128-2 in apoptosis of CLP

**Figure 7:**
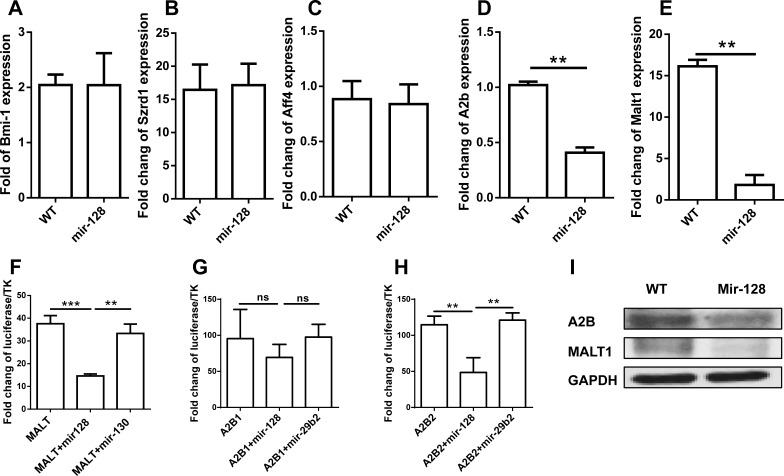
Identification of target genes of miR-128-2 Real-time PCR assay was adopted to evaluate the expression of Bmi-1, Szrd1, Aff4, A2b, and Malt1 **A.**- **E.**; Luciferase assays were conducted to measure the inhibition of miR-128-2 on the expression of MALT1 **F.** and A2B **G.** and **H.**. Protein expression levels of A2B and MALT1 were detected by Western blot **I.**, with GAPDH as loading reference. The data represent three repeats. **P < 0.01, ***P < 0.001, ns: no significant difference.

To explore the underlying mechanisms of miR-128-2 function in the apoptosis and differentiation of CLP, we used miRWalk software to predict the target genes of miR-128 and select those that can be predicted by more than five programs. The obtained gene list was further intersected with genes related to apoptosis and B cell development according to GO analysis. Finally, we selected BMI-1, SZRD1, AFF4, A2B, and MALT1 for further investigation. First, we detected the expression of these genes in CLP by real-time PCR. Figure 7A-7E illustrates that the mRNAs expression levels of Bmi-1, Szrd1, and Aff4 between WT and miR-128-2-overexpressed CLP did not differ, although the mRNA expression levels of A2B and MALT1 were obviously downregulated in miR-128-2-overexpressed CLP compared with those in WT CLP. To determine whether miR-128-2 directly targets A2B and MALT1 for repressing gene expression, we first conducted the luciferase report assay to verify whether miR-128-2 can bind to the 3′-UTR of A2B and MALT1. Plasmids encoding miR-130 or miR-29b2 preserved in our laboratory were used as controls. Luciferase assays showed that miR-128-2 could bind to the 3′-UTR of A2B and MALT1 and downregulate the expression of luciferase (Figure 7F-7H). Western blot experiments demonstrated that the protein levels of A2B and MALT1 were both significantly lower in miR-128-2-overexpressed B220^+^IgM^−^ preproB cells than those in WT cells (Figure 7I). These results strongly suggested that A2B and MALT1 were the target genes of miR-128-2, which indicated that these two genes might be involved in the decreased apoptosis of CLP and development of CLP to preproB cells.

### MiR-128-2 affects CLP apoptosis through the ERK and p38 MAPK signaling pathways

**Figure 8:**
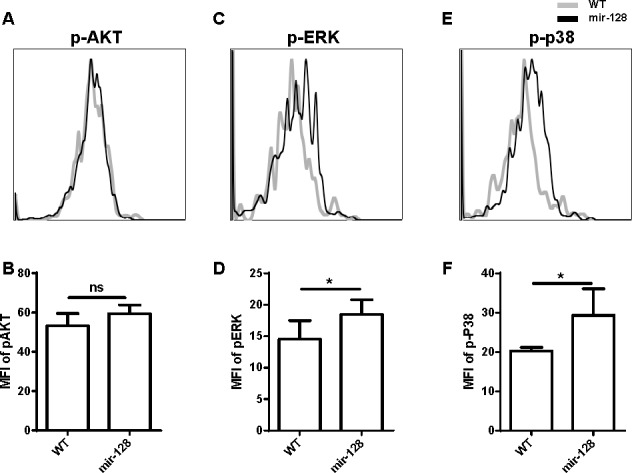
Phosphorylation of ERK and p38 MAPK was enhanced in miR-128-2-overexpressed CLP compared with those in WT CLP The intracellular staining were conducted followed by FACS analysis showed as overlap histogram, the mean fluorescence intensity (MFI) were analyzed by FlowJo software. The data represent three repeats. ns: no significant difference, *P < 0.05.

We used Ingenuity Pathways Analysis software (IPA, Ingenuity Systems, http://www.ingenuity.com) to further investigate through which pathways A2B, MALT1, or other potential targets are involved in the apoptosis of miR-128-2-overexpressed CLP, as well as to analyze the signaling pathways that may be mediated by them. As shown in Supplementary Figure 10, miR-128-2 may be involved in the AKT, p38 MAPK, NF-κb, and Fos pathways. Intracellular staining revealed that phosphorylation of ERK and p38 MAPK was obviously enhanced in miR-128-2-overexpressed CLP compared with that in WT CLPs (Figure 8). This result suggested that miR-128-2 overexpression resulted in hyperactive ERK and p38 signaling pathways, which may cause decreased apoptosis of CLPs. However, the mechanism by which the combination of A2B and MALT1 altered the phosphorylation of ERK and p38 remains unclear.

## DISCUSSION

MiR-128 is highly expressed in the brain and has been reported to play key roles in the development of the nervous system and maintenance of its normal physical functions [26]. Moreover, abnormal expression levels of miR-128 were detected in several cancer patients. Numerous studies have demonstrated that miR-128 can regulate the proliferation, differentiation, and apoptosis of various tumor cells [27]. However, whether miR-128 is also involved in lymphocyte development is largely unknown. In the present study, we found that miR-128 was differentially expressed among various immunocytes, which indicated that miR-128 may also be involved in the development of lymphocytes. By establishing miR-128-2 chimera and TG mice models, we found that ectopic expression of miR-128-2 could impair B cell development. Although miR-128-2 was significantly differentially expressed in different subsets of T cells (Figure 1B), these subsets were not affected in miR-128-2-overexpressed chimera mice or in miR-128-2 sponge chimera mice (Supplementary Figure 3 and data not shown). Thus, miR-128-2 for T cell development may be redundant. However, this hypothesis for the miR-128-2 knockout mice model requires further confirmation.

By screening the checkpoints of B cell development, we found that overexpression of miR-128-2 blocked CLPs from developing into preproB cells. B cells develop from HSCs in a highly ordered multistep process. HSCs yield MPPs, which subsequently lead to lymphoid-primed MPPs and CLPs. CLPs generate B220^+^ preproB cells, the earliest stage of the B cell lineage. The B cell development pathway has been thoroughly investigated and characterized, revealing important growth factors and regulatory interactions. The TFs E2A and EBF1 direct CLPs into the B cell developmental pathway. Together with PAX5 and Ikaros, these factors initiate the progressive steps of V(D)J recombination and expression of accessory proteins required for the display of premature and mature B cell receptors [23]. Since Ambros and Ruvkun identified lin-4 as a small RNA that controls larval development in *Caenorhabditis elegans* through the negative regulation of lin-14 in the last two decades [7, 8], thousands of miRNAs have been identified in nearly 200 species and are recognized as a previously unforeseen regulatory layer of gene regulation critical to a plethora of biological processes [6]. The first miRNA reported to have a role in B cell differentiation was miR-181a [11]. In this study, miR-181a was identified as an miRNA differentially expressed in T and B cells, and the ectopic expression of miR-181a generated a substantial increase in the generation of B cells both *in vitro* and *in vivo* [11]. However, miR-181a was shown to downregulate the threshold of TCR signaling in thymocytes [28]. Subsequently, miR-17-92 [29], miR-34a [18], and miR-150 [12] were reported to regulate proB cell to preB cell development, how and whether miRNAs can affect CLPs to develop into B cells are largely unclear. Our *in vivo* and *in vitro* experiments showed that miR-128-2 may impair B cell development by blocking CLP to further develop into preproB cells. This paper is the first to report that miRNA was also involved in this important checkpoint of B cell development. The overexpression of miR-128-2 did not alter the percentages of HSC and MPP, but increased the percentage of CLP and decreased the percentages of preproB, proB, immature B cells, and recirculating B cells. CLPs are a heterogeneous cell population that includes ALP and BLP. BLP is assumed to directly develop into B cells, whereas ALP develops into T cells or BLP [23]. We observed that the ratio of ALP and BLP did not change between WT and miR-128-2-overexpressed CLPs. The proliferation of miR-128-2-overexpressed CLPs also did not change compared with that of WT CLPs. However, apoptosis significantly decreased in miR-128-2-overexpressed CLP, which may result in the increase in percentage of CLP and block CLPs from further differentiating into preproB cells. Very few studies demonstrated the mechanisms of apoptosis of CLP. The present study may initiate a novel direction for the molecular regulation of CLP development. To further explain the molecular mechanism by which miR-128-2 affects the apoptosis of CLP, we attempted to seek for relevant targets of miR-128-2. Although some key TFs play pivotal roles in B cell development, such as RAG, AFF4, Bmi-1, IKZF3, RBPJ, BCL2L11, Dclrec, and PTEN, no difference was observed in their expression levels between WT and miR-128-2-overexpressed B cells or CLPs (Figure 7 and data not shown). Nevertheless, luciferase assay and real-time PCR combined with Western blot suggested that MALT1 and A2B were the target genes of miR-128-2 in CLPs.

Hajiahmadi and colleagues found that A2BAR agonist NECA can induce ovarian cancer cell apoptosis via Bax/Bcl-2 and caspase-3 [30]. Our study found that miR-128-2 could inhibit the apoptosis of CLP by targeting A2B. Hence, further investigations are necessary to determine whether the function of A2B in the apoptosis of CLP is through the same pathways as in ovarian cancer cells.

The specific feature of miRNAs is their ability to target many genes in one cell and target different genes in various cells, thereby performing subtle regulatory functions. MALT1, a human paracaspase operating downstream of BCL10, controls the catalytic activity of the canonical IKK complex and regulates the signaling of JNK and p38 MAP kinases. The absence of MALT1 reduces the frequency of marginal zone B cells and CD5^+^ B1 cells, but does not change the number of T and NK cells. Our study also found that miR-128-2 may also target MALT1 in CLPs in combination with A2B to regulate CLP development through the ERK and p38 MAPK pathways. The phenotypes of miR-128-2 TG mice were not completely consistent with MALT1-deficient mice, which may be due to miR-128-2 targeting multiple genes in CLP [31, 32].

In summary, our study suggested that mir-128-2, which is highly expressed in progenitor and immature lymphocytes, regulated CLP to develop into preproB cells. MiR-128-2 overexpression could inhibit the apoptosis of CLP by regulating the ERK and P38MAPK pathways via targeting the A2B and MALT1 genes. Our further studies will focus on whether abnormal expression of miR-128-2 is associated with certain lymphoid diseases in clinical settings.

## MATERIALS AND METHODS

### Mice

Six- to eight-week-old C57/BL6 mice were purchased from Shanghai SLAC Laboratory Animal Co. (Shanghai, China). The miR-128-2 TG mice used in this study were generated by Cyagen Bioscience, Inc(Guangzhou, China). All mice were maintained in a barrier facility at Soochow University. All animal experiments were approved by the Institutional Animal Care and Use Committee of Soochow University.

### Preparation of miR-128-2 chimera and TG mice models

The plasmids pMSCV_GW_RfA_PGK_EGFP-miR-128-2, pMSCV_GW_RfA_PGK_EGFP-miR-130, and pMSCV_GW_RfA_PGK_EGFP-miR-29b2 (hereafter called pMSCV-miR-128-2, pMSCV-miR-130, and pMSCV-miR-29b2, respectively) encoding mature miR-128-2, miR-130, and miR-29b2, respectively, were provided by Dr. Su (Shanghai Jiaotong University, Shanghai, China). Retroviral supernatant was generated using standard procedures after calcium phosphate transfection of pMSCV-128-2 and pCL-ECO viral packaging construct into 293T cells. To enrich hematopoietic stem/progenitor cells, donor mice were injected i.p. with 5mg of 5-fluorouracil 5days before BM harvest. BM cells were collected by flushing the tibia and femur with PBS/1% FBS, and red blood cells were lysed with ACK lysis buffer. BM cells were infected with retrovirus as in a previously described protocol [11]. Infected cells were resuspended in PBS and then injected i.v. into lethally irradiated (8.5Gy) recipient mice to establish the miR-128-2 chimera mice model. After two to four months, some immune cells of the mice were harvested for use in the experiments.

The generation of miR-128-2 TG mice pMSCV-128-2 plasmids was first linearized by HindIII restriction enzyme digestion and then micro-injected into ES cells with C57/BL6 background. The micro-manipulated ES cells were implanted into pseudocyesis C57/BL6 mice (Cyagen Biosciences Inc., Guangzhou, China). Positive offspring mice were bred with other mice from the same founder. Six- to eight-week-old miR-128-2 TG mice were used for the experiments.

### Antibodies

The following antibodies were purchased from Biolegend Inc. (San Diego, CA, USA): anti-CD3-pacific blue (17A2), anti-CD4-pacific blue (GK1.5), anti-NK1.1-pacific blue (PK136), anti-CD11b-pacific blue (M1/70), anti-CD11c-pacific blue (N418), anti-Ter-119-pacific blue (Ter-119), anti-CD8-pacific blue (53-6.7), anti-B220-pacific blue (RA3-61B2), anti-CD4-PE (GK1.5), anti-CD4-FITC (GK1.5), anti-CD8-APC (53-6.7), anti-CD8-Pecy7 (53-6.7), anti-CD23-PECy7 (B3B4), anti-CD21/CD35 (CR2/CR)-PerCP/Cy5.5 (7E9), anti-Gr1-Pecy7 (RB6-8C5), Anti-IAb-FITC (KH74), anti-B220-APC-Cy7 (RA3-61B2), anti-CD117-PE-Cy7 (2B8), anti-PDCA1-APC (927), anti-CD135 (flt3)-APC (A2F10), anti-CD25-PE (3C7), anti-CD11C (N418), anti-IgD-PerCP/Cy5.5 (11-26c.2a), anti-Sca1-APC (D7), anti-IgM-PE (RMM-1), anti-CD16/32-APC/Cy7(93), anti-CD127 (IL-7R)-PerCP/Cy5.5 (SB/199), anti-CD93 (AA4.1)-PE (AA4.1), anti-CD117 (c-kit)-PE/Cy7 (2B8), and anti-CD34-PE (MEC14.7). Anti-human/mouse phospho-ERK1/2 (T202/Y204)-APC (MILAN8R), anti-human-mouse phospho-p38 (T180/Y182)-APC (4NIT4KK), and anti-human/mouse phosphor-AKT (S473)-APC (SDRNR) were purchased from eBioscience (San Diego, CA, USA).

### Cell staining and flow cytometry

Single-cell suspensions of BM and spleen from mice were prepared, blocked with antibody against Fc receptors, and stained with the above antibodies in different combinations [Lin^−^IL-7R^+^c-kit^hi^Sca1^+^, Lin^−^IL-7R^+^ckit^+^Sca1^−^, Lin^−^IL-7R^+^ckit^int^Sca1^+^, Lin^−^IL-7R^+^ckit^int^Sca1^+^ly6D^−^, Lin^−^IL-7R^+^ckit^int^Sca1^+^ly6D^+^, B220^+^IgM^+^CD43^hi^, B220^+^IgM^+^CD43^int^, B220^+^IgM^+^CD43^−^, CD11b^+^Gr1^+^, CD11c^+^IAb^+^, and CD11b^−^B220^+^CD11C^+^PDCA1^+^ represent HSCs, multipotent progenitor (MPP) cells, CLP, all-lymphoid progenitor (ALP), B cell-biased lymphocyte progenitor (BLP), preproB, proB, preB, myeloid-derived suppressor cells (MDSCs), cDC, and pDC, respectively]. Lineage antibody cocktails included pacific blue-labeled anti-CD3, anti-CD4, anti-CD8, anti-B220, anti-Gr1, anti-CD11b, anti-NK1.1, and anti-Ter119 antibodies. For intracellular staining, some surface markers were stained and the cells were fixed, permeabilized, and stained with phosphor-AKT, phosphor-ERK, or phosphor-p38MAPK antibodies. For apoptosis assay, BM or spleen cells were first stained with relative surface markers, washed, and stained with annexin V in annexin V binding buffer. After staining, the cells were collected by BD FACS AriaIII or BD FACS CantoII (Franklin Lakes, NJ, USA). FACS data were analyzed using FlowJo software (Tree Star, Inc. Ashland, OR, USA).

### BrdU staining

For BrdU incorporation assay, each mouse was injected with 1mg of BrdU i.p. at 12 and 4h before sacrifice. The BM and spleen cells were harvested for staining of relative surface markers and subsequent intracellular BrdU according to the manufacturer's protocol. After staining, the cells were collected by BD FACS CantoII (Franklin Lakes, NJ, USA). FACS data were analyzed using FlowJo software (Tree Star, Inc. Ashland, OR, USA).

### Real-time PCR assay

Total RNAs were extracted from different tissues (BM, thymus, and spleen) and purified cells, including single-positive T cells (CD4^+^, CD8^+^), DP (CD4^+^CD8^+^), DN (CD4^−^CD8^−^), CLP, preproB, immature B, and recirculating B cells, or 293T cells using RNAiso Plus reagent (TAKARA Biotechnology Co. Ltd., Dalian, China). To detect miR-128-2 expression, total RNAs were reversed using MMLV reverse transcriptase with miR-128-2 specific RT primer 5′-CTC AAC TGG TGT CGT GGA GTC GGC AAT TCA GTT GAG AAA GAG AC-3′. The resultant cDNA was then used as a template to perform real-time PCR using a Roche real-time PCR kit with specific PCR primers: F: 5′-AAC ACT CCA GCT GGG TCA CAG TGA ACC GGT CT-3′, R: 5′-CTC AAC TGG TGT CGT GGA-3′. To detect the expression of other genes including BMI-1, SZRD1, AFF4, A2B, and MALT1, total RNAs were reversed using MMLV reverse transcriptase with Oligo (dT). Transcripts were quantified by real-time PCR and normalized to the amount of GAPDH mRNA expression. The PCR primers are listed in Supplementary Table 1.

### Luciferase activity assay

The oligonucleotides containing sequences predicted as the binding sites of miR-128-2 in A2B and MALT1 cDNA were synthesized (Supplementary Table 1), annealed, and cloned downstream of CMV-driven firefly luciferase cassette in pMIR-REPORT vector (Ambion Co., Waltham, MA USA). To validate miRNA targets, approximately 10^5^ 293T cells per well in a 24-well plate were transiently transfected with 0.3μg of each firefly luciferase reporter construct, 0.1μg of Renilla luciferase TK vector, and 0.6μg of pMSCV-miR-128-2 or control vector of pMSCV-miR-130 or pMSCV-miR-29b2. Renilla luciferase TK vector was used to normalize transfection efficiency. At 24h after transfection, firefly and Renilla luciferase activities were assayed (Promega Co., Madison, WI, USA). Firefly luciferase activity was normalized to Renilla luciferase activity, which is the internal control for transfection. The relative units represent firefly luciferase activity/Renilla luciferase activity.

### Northern blot analysis

Total RNAs were extracted from 293T or pMSCV-miR-128-2-transfected 293T cells for the detection of miR-128-2 expression using a highly sensitive miRNA Northern blot assay kit (Signosis, Inc., Sunnyvale, CA, USA). In brief, 15% TBE-Urea gel was pre-run at 60V for approximately 30min, and 5–20μg of RNA was loaded at 60V. RNAs were transferred to a membrane at 60V for 1h, followed by UV crosslinking for 10min. The membrane was then hybridized with biotin-labeled miRNA probes and streptavidin-horseradish peroxidase (HRP) conjugate. The membrane was exposed using X-ray film after adding the substrate. 18S and 28S RNA were used as loading reference.

### Western blot analysis

Single-cell suspensions from BM of wild-type (WT) and miR-128-2 TG mice were prepared for staining with specific fluorescence-conjugated antibodies. PreproB (B220^+^IgM^−^) and CLP (Lin^−^ckit^int^Sca1^+^) cells were sorted using FACS ArrayII. Sorted cells were lysed with RIPA lysis buffer (Beyotime Institute of Biotechnology, Shanghai, China). Equal concentrations of protein were separated on a denaturing sodium dodecyl sulfate–10% polyacrylamide gel and then transferred to nitrocellulose by electroblotting. Proteins were detected with a 1:1000 dilution of rabbit anti-MALT1 (#2494s, Cell Signaling Inc., Danvers, MA, USA) or goat anti-A2B (R-20) (#Sc-7507, Santa Cruz Biotechnology Inc., Dallas, TX, USA) and a 1:5000 dilution of HRP-conjugated anti-rabbit antibodies (#7704, Cell Signaling Technology, MA, USA) or HRP-conjugated donkey anti-goat antibodies (#Sc2020, Santa Cruz Biotechnology Inc., Dallas, TX, USA). GAPDH (#14C10, Cell Signaling Inc., Danvers, MA, USA) was used as loading reference. HRP was detected with SuperSignal West Dura Extended Duration Substrate (Thermo Scientific, Waltham, MA, USA).

### B cell development *in vitro*

Single-cell suspensions from BM of WT or miR-128-2 TG mice were prepared and then stained with anti-IL-7R-PE, anti-ckit-pecy7, anti-Sca1-APC, and lineage antibodies (pacific blue-labeled anti-CD3, CD4, CD8, CD11c, CD11b, B220, NK1.1, Gr1, and Ter-199 antibodies). After washing three times, the labeled cells were sorted for CLP (Lin^−^ IL7R^+^ckit^int^Sca1^+^) using FACS ArrayII (Franklin Lakes, NJ, USA). The sorted CLP cells were cultured on a confluent layer of OP9 stromal cells in 24-well plates containing DMEM with 15% FCS, IL-7 (5ng/mL), SCF (10ng/mL), and Flt3-L (10ng/mL). After 5days, suspension cells were gently harvested and the cell number was counted. The remaining cells were stained with anti-B220 and anti-CD19 antibodies to analyze the percentage of B cells using FACS CantoII (Franklin Lakes, NJ, USA).

### Statistical analysis

Statistical analysis was performed using Student's *t*-test. P values less than 0.05 were considered statistically significant.

## SUPPLEMENTARY MATERIAL TABLE AND FIGURES


