# Supplementary material for: MiR-128-2 inhibits common lymphoid progenitors from developing into progenitor B cells
Source: Oncotarget. 2016 Mar 17;7(14):17520–31. doi: 10.18632/oncotarget.8161 (PMC4951230; doi:10.18632/oncotarget.8161)
Supplement: Supplementary file 1 [file oncotarget-07-17520-s001.pdf]

MiR-128-2 inhibits common lymphoid progenitors from developing into progenitor B cells

Supplementary Material

Supplementary Table 1 oligonucleotides used for real time PCR and plasmid construction

Name	Sequence (5'-3')
BMi-1 F	ATGTATTGTACGTTACTTGGAG
BMi-1 R	GTCTTCATTGGAGCCATTGG
Szrd1 F	GAGTATGCAGAGGCTCGGAG
Szrd1 R	ACGGCTGTCTGATCACATTGC
Aff4 F	GCAACATGAACCGTGAAGACC
Aff4 R	ATCGATCTGTCTCCTATGTAATC
Malt1 F	TAGGATTTGCCGCAGAGTTC
Malt1 R	AGTCTAGTGTAGAGGCAGTG
A2bF	CCTTTGCCATCACCATCAGC
A2bR	AGATATCGGTCGACAGCCAC
Malt1-luciferase F	CTAGTCAGACATATATGCAGTCAGGACACTGTGTAAATAAA
Malt1-luciferase R	AGCTTTTATTTACACAGTGTCCTGACTGCATATATGTCTGA
A2B-luciferase1F	CTAGTCAACTGGCCGATCCTCACTGTGAAAGACAGCTGCACCTCCCAAGCA
A2B-luciferase1R	AGCTTGCTTGGGAGGTGCAGCTGTCTTTCACAGTGAGGATCGGCCAGTTGA
A2B-luciferase2F	CTAGTGTGGTAGAAAATGACTGAAACTTACCTTACTGTGAAACACTGTGAACA
A2B-luciferase2R	AGCTTGTTCACAGTGTTTCACAGTAAGGTAAGTTTCAGTCATTTTCTACCACA

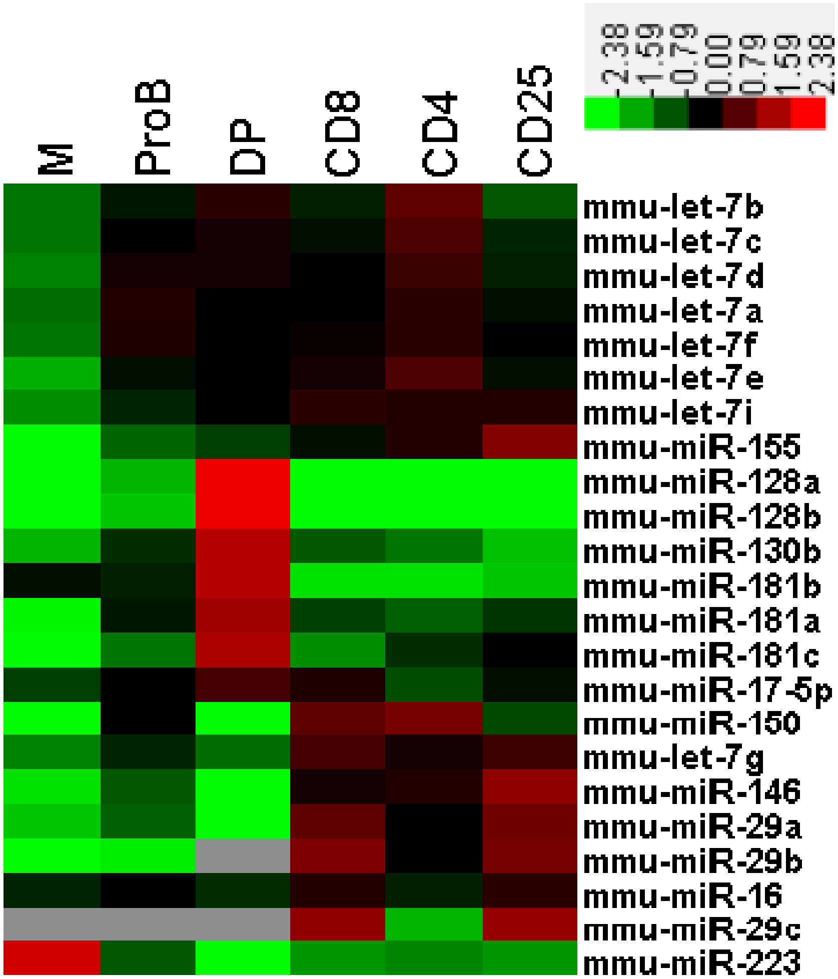

Supplementary Figure 1. MiRNA expression profile in various immunocytes detected by microarray. Monocytes and proB cells were purified from BM; $CD4^+CD8^+$ double-positive thymocytes were purified from the thymus; $CD4^+$, $CD8^+$, and $CD4^+CD25^+$ cells were purified from splenocytes.

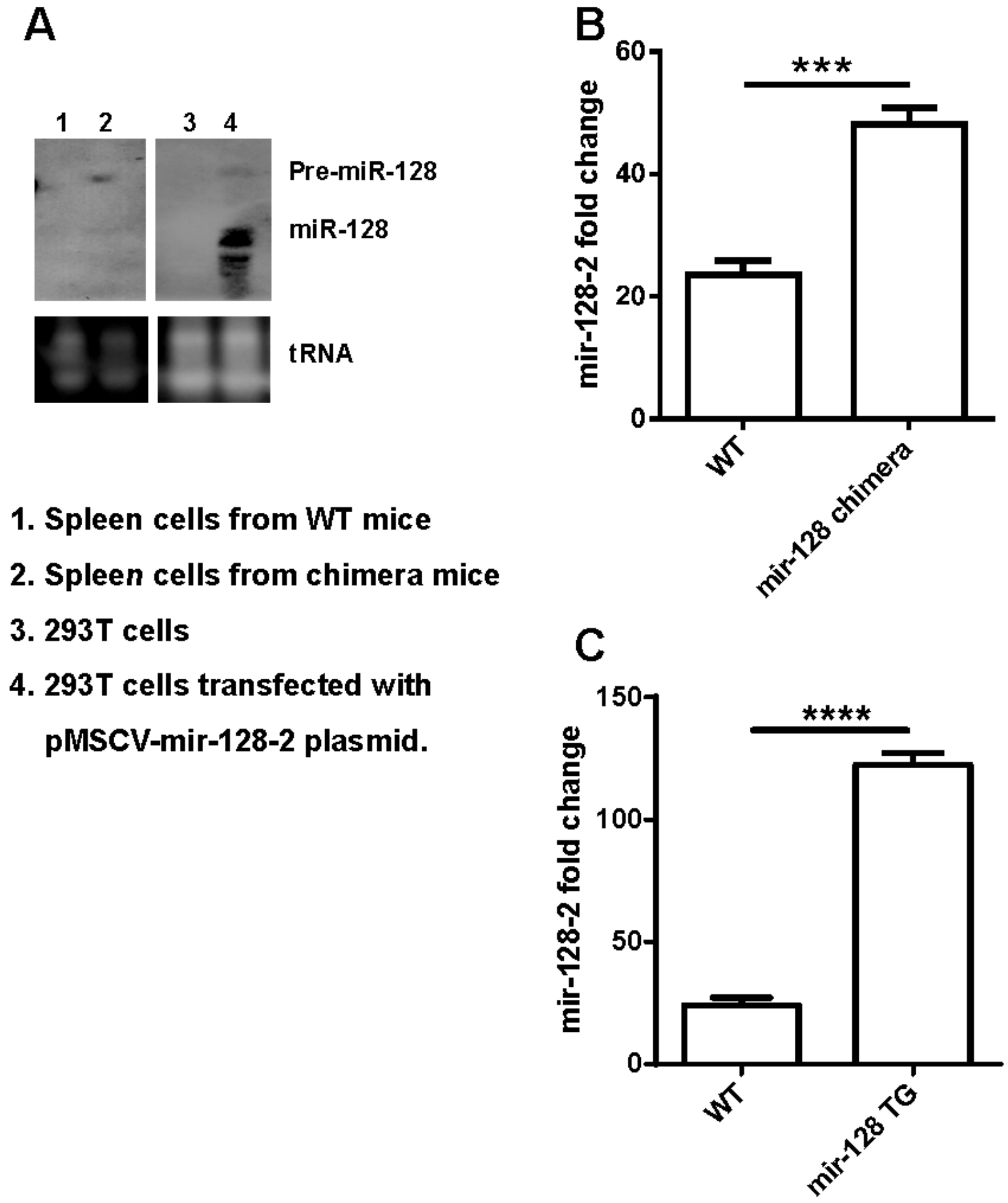

Supplementary Figure 2. The expression of miR-128-2. (**A**) The expression of miR-128-2 in spleen cells from WT and miR-128-2 chimera mice, or 293T cells and pMSCV-miR-128-2 transfected 293T cells detected by Northern-blot. The expression of miR-128-2 in spleen from WT, chimera mice (**B**) or TG mice (**C**) were measured by real time PCR. ***P<0.001, ****P<0.0001. The data represent three repeats.

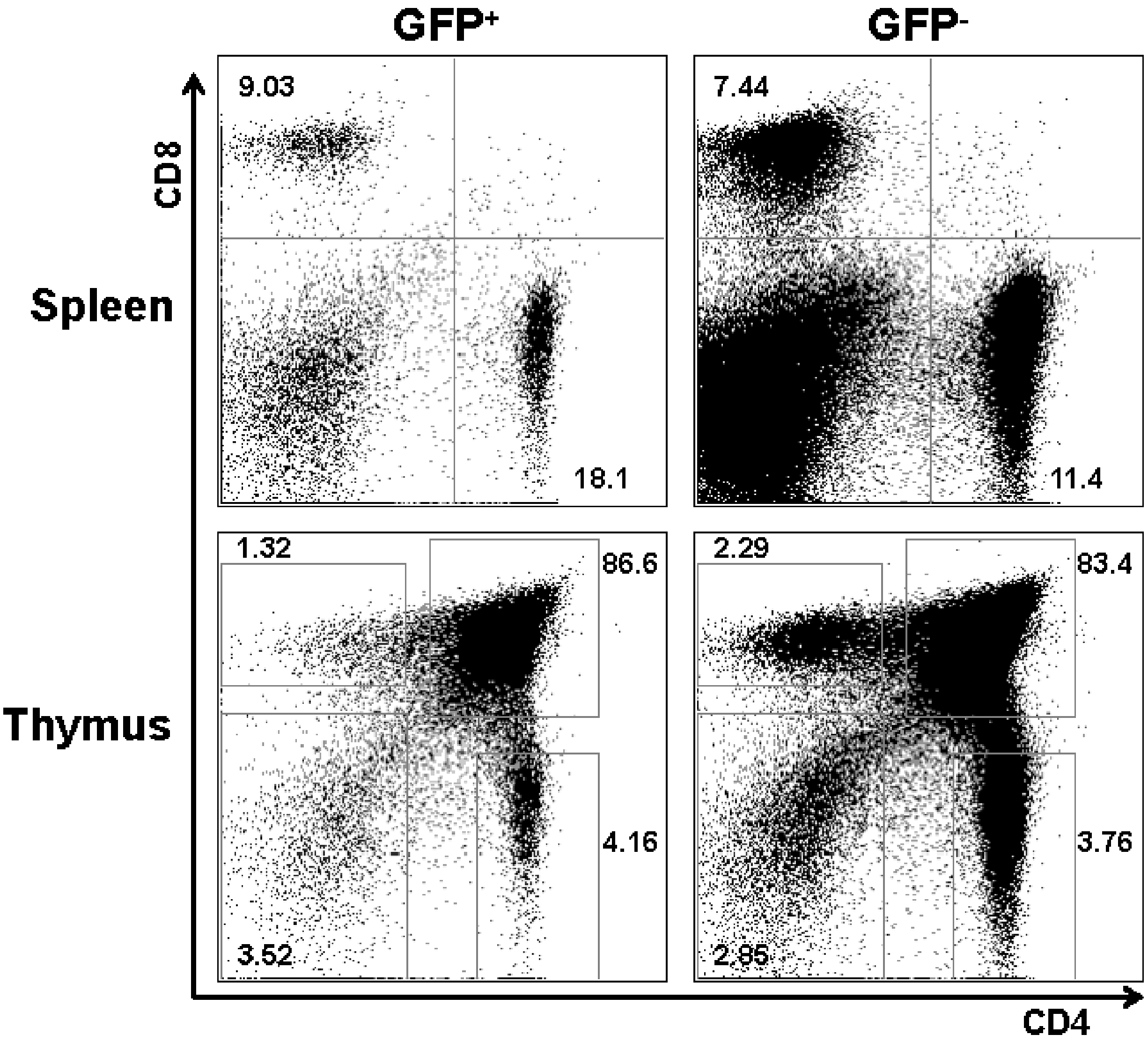

Supplementary Figure 3. Overexpression of miR-128-2 does not significantly alter the percentages of the T cell subset in the spleen and thymus. The data represent five repeats.

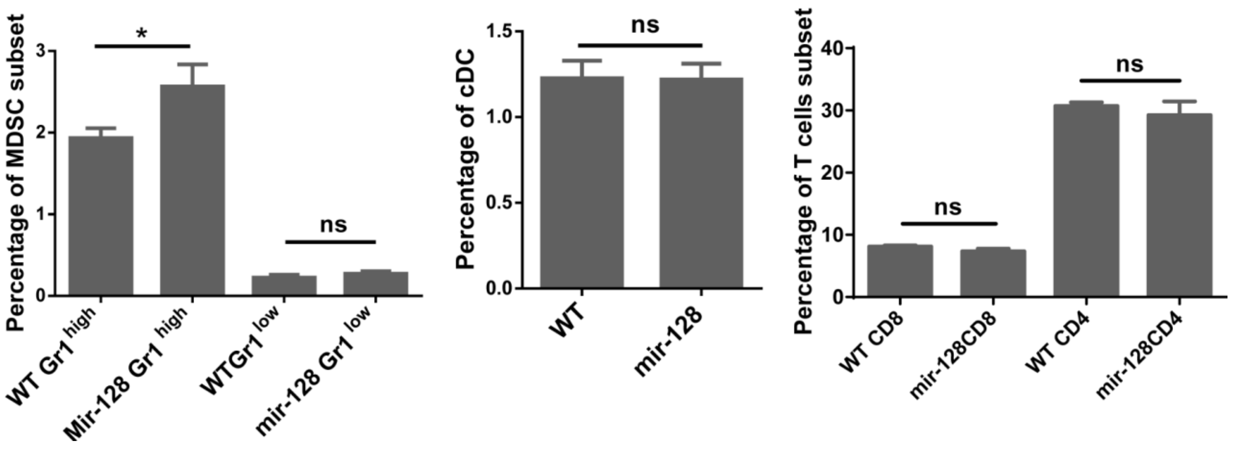

Supplementary Figure 4. Percentages of MDSC, cDC, and T cells were not altered in miR-128-2-overexpressed TG mice. BM cells from WT or TG mice were stained with anti-Gr1/anti-CD11b antibodies and sorted $CD11b^{+}Gr1^{high}$, $CD11b^{+}Gr1^{low}$ cells as MDSC. Spleen cells from WT or TG mice were stained with anti-CD11c/anti-IAb antibodies or anti-CD4/anti-CD8 antibodies and sorted $CD11c^{+}IAb^{+}$ cells as cDC, $CD4^{+}CD8^{-}$, $CD4^{-}CD8^{+}$ as CD4 or CD8 T cells, respectively. The data represent three repeats. ns: no significant difference, *P<0.05.

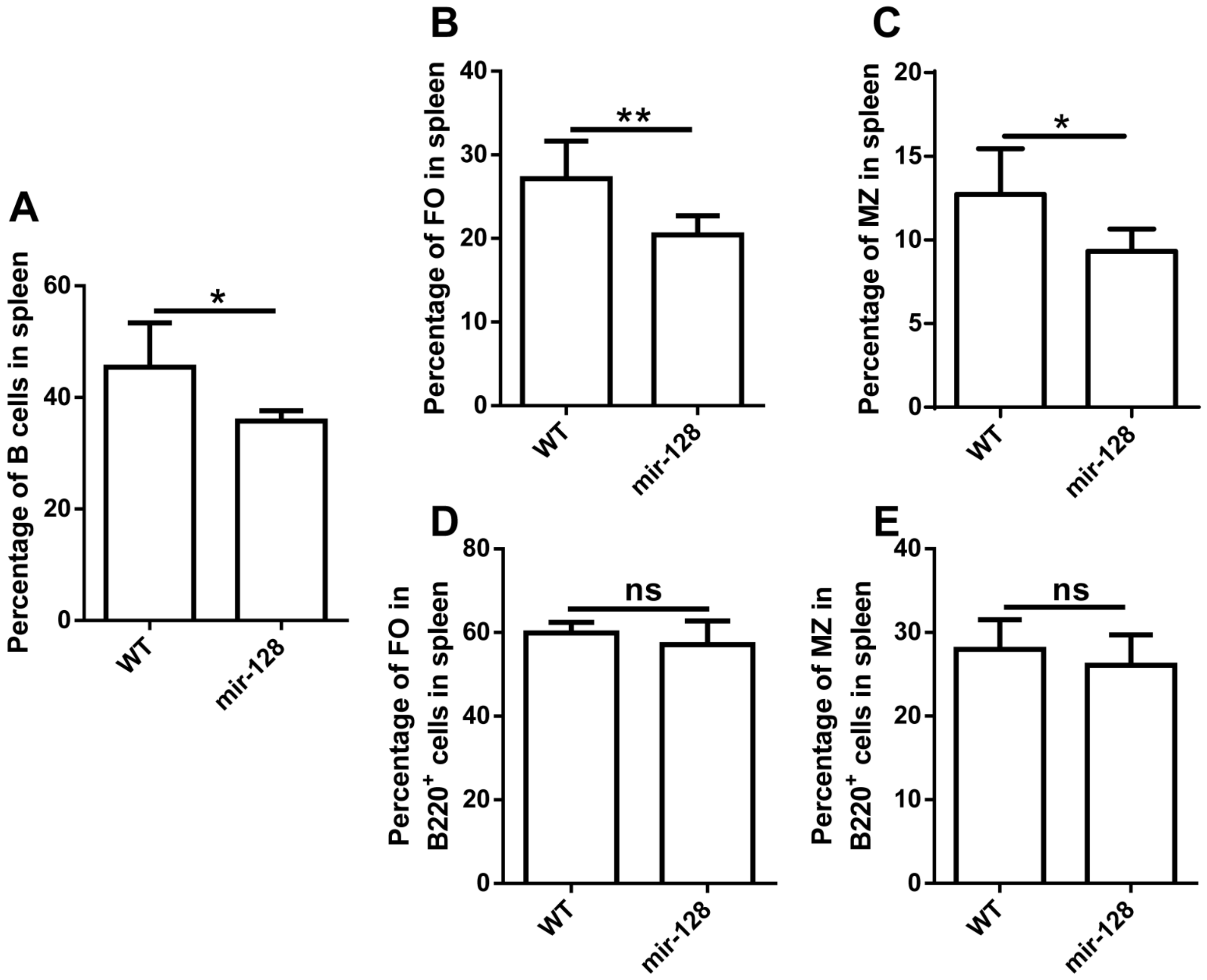

Supplementary Figure 5. Peripheral B cells were reduction in spleen of miR-128-2 over-expressed TG mice compared with those in WT mice. The spleen cells were stained with anti-B220/anti-AA4.1/anti-CD21/anti-CD23 antibodies. The percentages of total B cells ($B220^{+}$), MZ ($B220^{+}AA4.1^{-}CD21^{+}CD23^{low}$), FO($B220^{+}AA4.1^{-}CD21^{+}CD23^{high}$) were all decreased (**A, B, C**). The percentages of FO or MZ in $B220^{+}$ cells were not significantly changed (**D, E**). The data represent three repeats. *P<0.05, **P<0.01, ns: no significant difference.

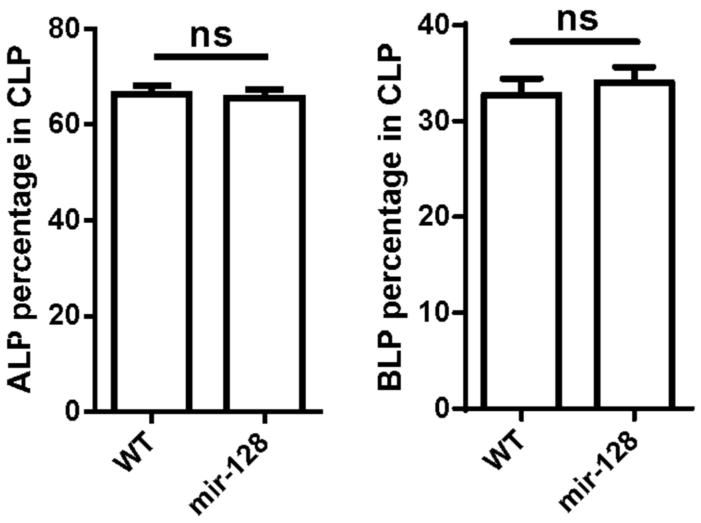

Supplementary Figure 6. Ratios of ALP and BLP to CLP were not changed in BM of miR-128-2-overexpressed TG mice compared with those in WT mice. The BM cells from WT or miR-128-2-over-expressed TG mice were stained with Lineage antibodies as described in M&M and c-kit, Sca1 and ly6D antibodies and analyzed by FACS. $Lin^{-}IL\text{-}7R^{+}ckit^{int}Sca1^{+}ly6D^{-}$ and $Lin^{-}IL\text{-}7R^{+}ckit^{int}Sca1^{+}ly6D^{+}$ cells represent ALP and BLP, respectively. The data represent five repeats. ns: no significant difference.

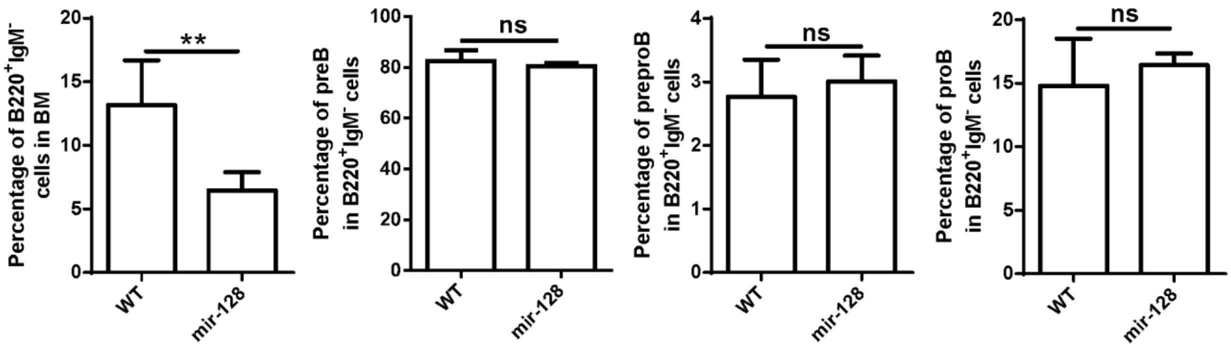

Supplementary Figure 7. The B cells subset in BM detected by FACS. BM cells from WT or miR-128-2 over-expressed TG mice were stained with relative antibodies followed by FACS analysis to evaluate the ratios of preproB ($B220^{+}IgM^{-}CD43^{high}$), proB ($B220^{+}IgM^{-}CD43^{int}$) and preB cells ($B220^{+}IgM^{-}CD43^{-}$) in $B220^{+}IgM^{-}$ BM cells . The data represent three repeats. **P<0.01, ns: no significant difference.

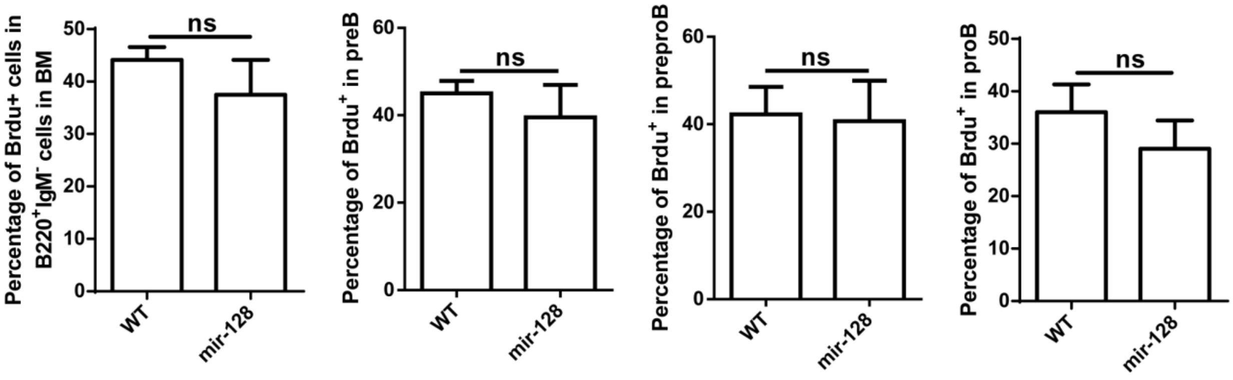

Supplementary Figure 8. The proliferation of B cells subset in BM evaluated by Brdu incorporation assay. BM cells from WT or miR-128-2 over-expressed TG mice were stained with surface marker (B220, CD43, IgM) and anti-Brdu antibody followed by FACS analysis to evaluate the proliferation of $B220^{+}IgM^{-}$ BM cells, preproB ($B220^{+}IgM^{-}CD43^{high}$), proB ($B220^{+}IgM^{-}CD43^{int}$) and preB cells ($B220^{+}IgM^{-}CD43^{-}$). The data represent three repeats. ns: no significant difference.

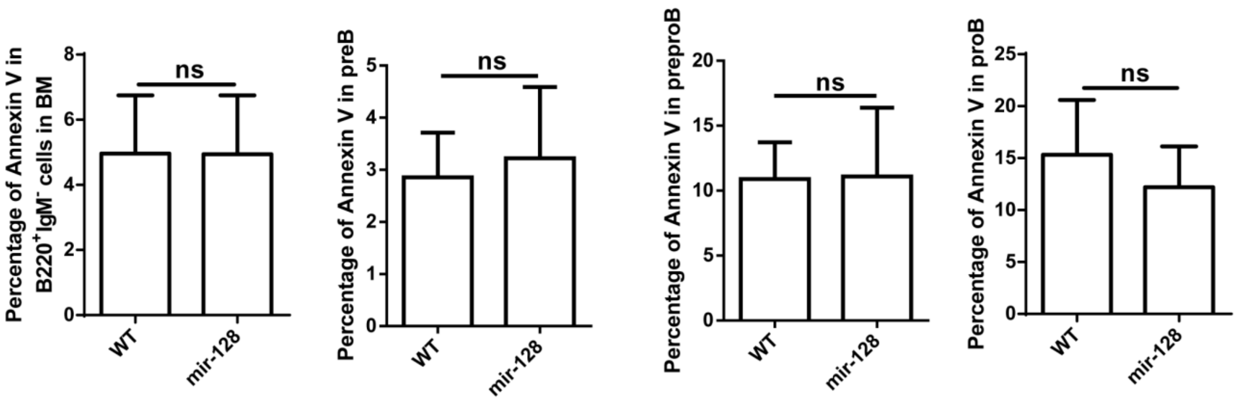

Supplementary Figure 9. The apoptosis of B cells subset in BM evaluated by Annexin V staining. BM cells from WT or miR-128-2 over-expressed TG mice were stained with antibodies against B220, IgM, CD43 and Annexin V followed by FACS analysis to measure to apoptosis of $B220^{+}IgM^{-}$ BM cells, preproB ($B220^{+}IgM^{-}CD43^{high}$), proB ($B220^{+}IgM^{-}CD43^{int}$) and preB cells ($B220^{+}IgM^{-}CD43^{-}$). The data represent three repeats. ns: no significant difference.

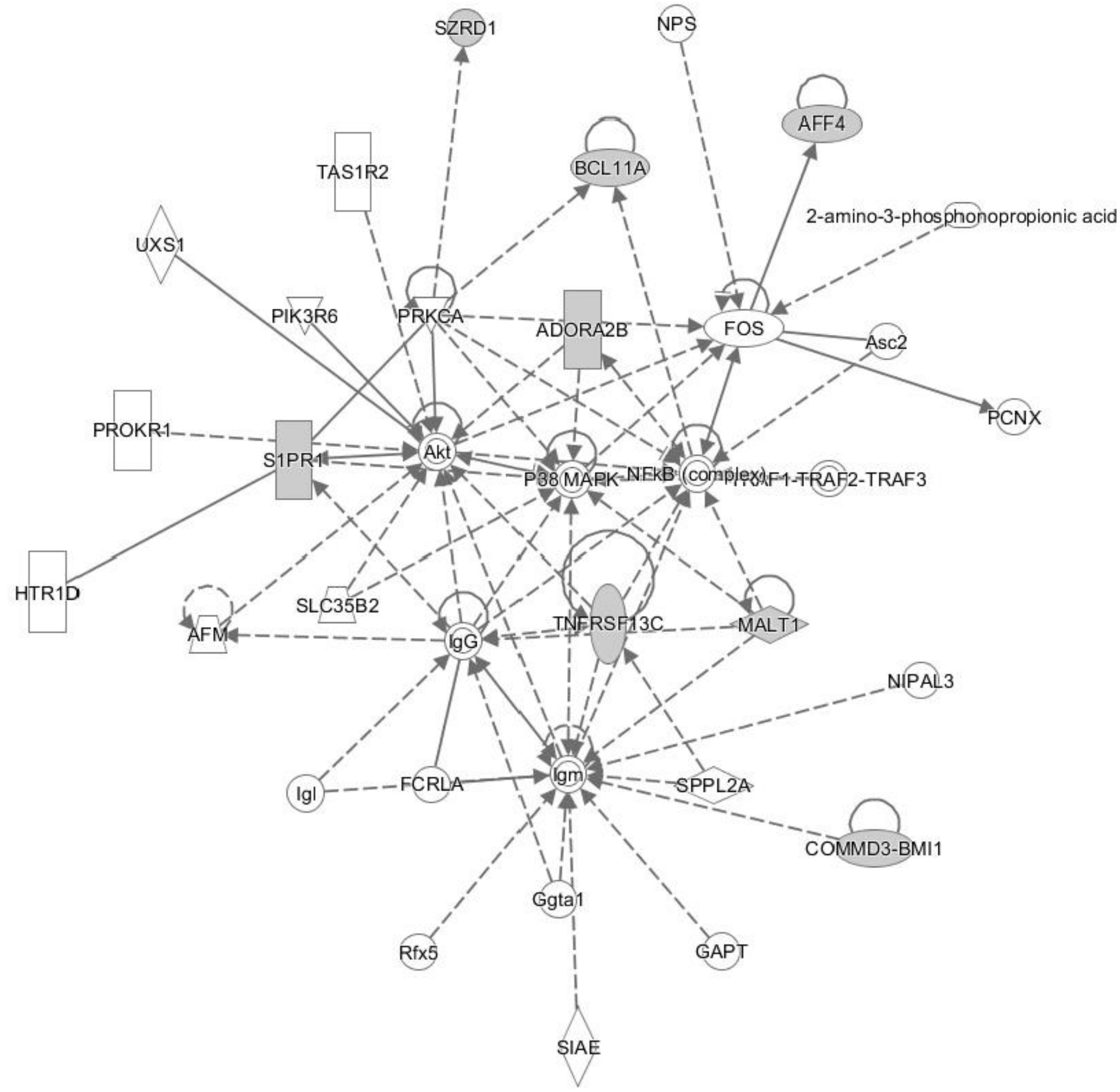

Supplementary Figure 10. Genes among the targets of miR-128 predicted by mIRwalk software that are involved in B cell apoptosis were submitted to analyze potential pathways.
